# The clinical significance of SWI/SNF complex in pancreatic cancer

**DOI:** 10.3892/ijo.2012.1723

**Published:** 2012-11-30

**Authors:** MASAKATSU NUMATA, SOICHIRO MORINAGA, TAKUO WATANABE, HIROSHI TAMAGAWA, NAOTO YAMAMOTO, MANABU SHIOZAWA, YOSHIYASU NAKAMURA, YOICHI KAMEDA, SHINICHI OKAWA, YASUSHI RINO, MAKOTO AKAIKE, MUNETAKA MASUDA, YOHEI MIYAGI

**Affiliations:** 1 Department of Gastroenterological Surgery, Kanagawa Cancer Center, Asahi-ku, Yokohama, Kanagawa 241-0815;; 2 Molecular Pathology and Genetics Division, Kanagawa Cancer Center, Asahi-ku, Yokohama, Kanagawa 241-0815;; 3 Departments of Pathology, Kanagawa Cancer Center, Asahi-ku, Yokohama, Kanagawa 241-0815;; 4 Hepatobiliary and Pancreatic Medicine, Kanagawa Cancer Center, Asahi-ku, Yokohama, Kanagawa 241-0815;; 5 Gastroenterological Center, Yokohama City University Medical Center, Minami-ku, Yokohama, Kanagawa 232-0024;; 6 Department of Surgery, Yokohama City University, Kanazawa-ku, Yokohama, Kanagawa 236-0004, Japan

**Keywords:** pancreatic cancer, the SWItch/sucrose non-fermentable complex, prognostic factor

## Abstract

Chromatin remodeling factors have been the subject of great interest in oncology. However, little is known about their role in pancreatic cancer. The objective of this study was to clarify the clinical significance of the SWItch/sucrose nonfermentable (SWI/SNF) complex in patients with pancreatic cancer. A total of 68 patients with pancreatic cancer who underwent R0, 1 resection were enrolled. Cancer tissues were processed to tissue microarray, then stained immunohistochemically by using antibody of SWI/SNF components; BRM, BRG1, BAF250a, BAF180 and BAF47. The correlation of expression levels and clinicopathological outcomes were analyzed, followed by the multivariate analysis of prognostic factors for overall survival. The expression levels of the SWI/SNF components were categorized as low or high according to the median value of Histoscore. Statistical analysis revealed that BRM expression was related to tumor size, T factor, M factor, lymphatic invasion and stage BRG1 expression to histology and stage BAF180 expression to tumor size and BAF47 expression to lymphatic invasion, respectively. Multivariate Cox proportional hazard analysis showed that high BRM and low BAF180 expression levels were independent predictors of worse survival in patients with pancreatic cancer. High BRM, and low BAF180 were also independent prognostic factors for poor survival in the subgroup with adjuvant gemcitabine. These results suggest that the specific cofactors of SWI/SNF chromatin remodeling complex certainly have roles in pancreatic cancer. High BRM, and low BAF180 are useful biomarkers for poor prognosis in pancreatic cancer.

## Introduction

Pancreatic cancer remains a leading cause of cancer deaths in the advanced nation (1,2). The overall 5-year survival rate is reported to be less than 5% (3). A reliable and clinically relevant prognostic biomarker which can stratify the disease is needed for developing new strategies.

It is a known fact that chromatin, highly condensed and dynamically structured, can be temporally rearranged so that specific genes can be expressed or repressed (4). Studies have shown that modification of chromatin structure is an essential step in gene regulation primarily mediated by chromatin remodeling proteins. Among these proteins, histone is known to play a dynamic role in the regulation of transcription (5–7). Often, transcription is also regulated by other cofactors, and the balance of chromatin remodeling activities may be crucial to ensure accurate responses to developmental or environmental cues and to prevent the transition of normal cells into cancer cells (8).

The SWItch/sucrose non-fermentable (SWI/SNF) complex is a major complex of adenosine triphosphate (ATP)-dependent chromatin remodeling factors and controls the transcriptional activity of a variety of genes involved in cellular growth and transformation by altering chromatin structure (9–13). SWI/SNF complex, originally identified in yeast, is composed of more than 10 characterized subunits (14,15) and human SWI/SNF complexes contain one of the two core ATPase subunits, BRM or BRG1 (13,16–18). Growing genetic and molecular evidence indicates that specific subunits of the SWI/SNF complex can act as tumor suppressors (6,19). However, there is no report on the relationship between SWI/SNF components expression and the clinical significance of pancreatic cancer. In this study, we investigated the expression levels of SWI/SNF components to clarify the clinical impact of SWI/SNF complex on pancreatic cancer.

## Materials and methods

### Patients and samples

The surgical specimens of pancreatic cancer tissue obtained from 68 patients were evaluated. All of the patients had undergone macroscopically curative resection (R0, 1) at Kanagawa Cancer Center between July 2006 and April 2010. The clinicopathological characteristics of these patients are shown in Table I. In all cases, archival hematoxylin and eosin-stained (H&E) slides of the primary tumor were retrieved and reviewed to confirm the pathological features as well as to select suitable tissue blocks for immunohistochemical analysis. Informed consent was obtained from each patient. The Ethics Committees of the Kanagawa Cancer Center approved the protocol before initiation of the study. We declare no conflicts of interest.

### Tissue microarrays and immunohistochemistry

Microarrays consisting of cores, each measuring 2 mm in diameter, were prepared from formalin-fixed paraffin-embedded tissue blocks of surgically removed primary tumors. Each tissue core of the primary tumor was sampled.

Immunohistochemical staining was performed using commercially available polyclonal rabbit, or mouse antibodies raised against BRM (Abcam Inc., Cambridge, MA), BRG1 (Santa Cruz Biotechnology Inc., Santa Cruz, CA), BAF250a (Santa Cruz Biotechnology Inc.), BAF180 (Sigma-Aldrich Inc., St. Louis, MO), BAF47 (Santa Cruz Biotechnology Inc.). Tissue microarray blocks were sectioned at a thickness of 4 *μ*m and mounted on pre-coated glass slides. The sections were de-paraffinized through a graded series of xylene and rehydrated through a graded series of alcohol to distilled water. Endogenous peroxidase was quenched with 3% hydrogen peroxide in methanol at room temperature. The sections were placed in a 95°C solution of 0.01 M sodium citrate buffer (pH 6.0) for 40 min for antigen retrieval. Normal goat serum (5%) was then applied for 15 min to block any non-specific protein binding sites. Primary polyclonal antibodies were applied for 1 h at room temperature at the following dilutions: anti-BRM at 1:250, anti-BRG1 at 1:200, anti-BAF250a at 1:100, anti-BAF180 at 1:90 and BAF47 at 1:300. Immunoreactive proteins were detected using the Simple Stain MAX-PO (Multi).

All sections were counterstained with Mayer’s hematoxylin, and negative controls were included in each staining sequence. The intensity and global level of staining were scored semi-quantitatively for each tissue microarray by an investigator blinded to all of the clinicopathological variables. The global level of staining refers to the percentage of tumor cells that stained positively for an antibody within each tissue microarray at ×200 magnification using a light microscope.

### Scoring of immunohistochemical reactivity

**Figure 1.**
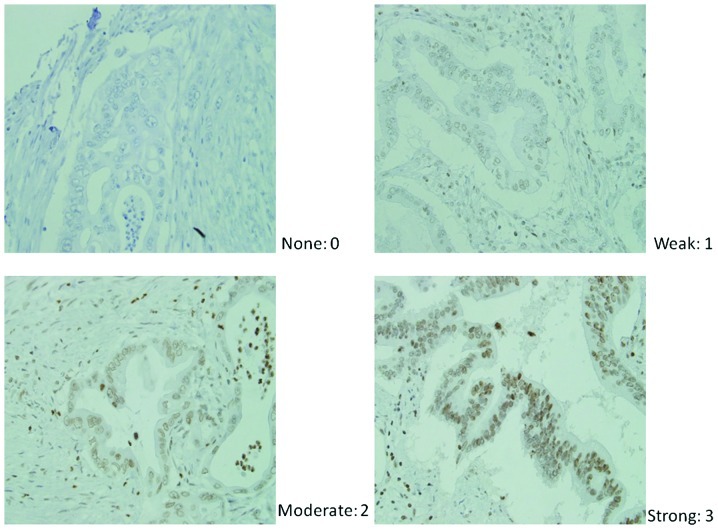
Histoscore (H-score) was calculated by a semi-quantitative assessment of both the intensity of staining (graded as: 0, non-staining; 1, weak; 2, median; or 3, strong using adjacent normal mucosa as the median) and the percentage of positive cells. The range of possible scores was from 0 to 300. Expression level of each component was categorized as low or high according to the median value of the H-score.

Immunohistochemical scoring was completed using the modified Histoscore (H-score) (20), which involves a semiquantitative assessment of both the intensity of staining (graded as: 0, non-staining; 1, weak; 2, median; or 3, strong using adjacent normal mucosa as the median) and the percentage of positive cells (Fig. 1). The range of possible scores was from 0 to 300. Expression level of each component was categorized as low or high according to the median value of H-score.

### Statistical analysis

The relationships between the expression level and the clinicopathological factors were evaluated with the χ^2^ test. The postoperative survival rate from the day of primary tumor resection was analyzed using the Kaplan-Meier method and any differences in the survival rates were assessed with the log-rank test. A Cox proportional-hazard model was used for the multivariate analyses. Differences were considered significant when P<0.05. The statistical analysis was performed using the PASW Statistics 18 (SPSS, Inc., Chicago, IL).

## Results

### Relation of SWI/SNF component expression to clinicopathological features

**Figure 2.**
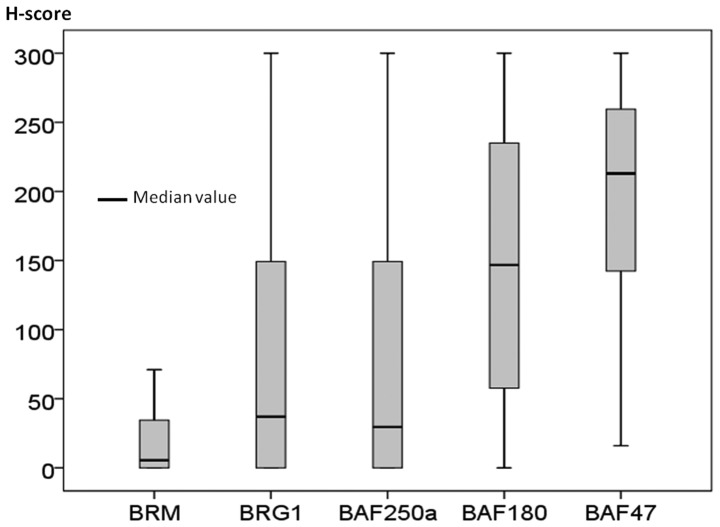
The distribution of the H-score is shown in the box plot. The horizontal bar shows the median value of each score.

The distribution of H-score is showed in Fig. 2. Expression level of the SWI/SNF components was categorized as low or high according to the median value of the H-score. Relations between the expression levels of each component and clinicopathological features were then examined. Factors implicating significant relations were tumor size, T factor, M factor, lymphatic invasion, and stage in BRM, histology and stage in BRG1, tumor size in BAF180, lymphatic invasion in BAF47, respectively (Table II).

### Analysis of prognostic factors in all patients

Univariate Cox regression analysis for overall survival in all patients showed that age, tumor size, histological type, M factor, curability of the surgery, and expression level of BRM as well as BAF180 were significant predictors (Table III). On multivariate Cox proportional hazard analysis, histology, expression level of BRM and BAF180 were significant independent predictors of overall survival in patients with pancreatic cancer (Table IV).

### Comparison of survival by the status of BRM and BAF180

**Figure 3.**
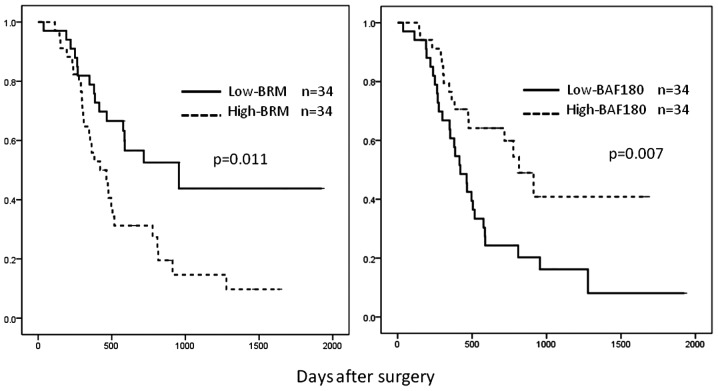
The survival curves were compared by Kaplan-Maier method by the expression level of BRM and BAF180. The statistical significance was evaluated using log-rank test.

The 5-year survival rate of high BRM patients was 9.8%, which was significantly worse than that of low BRM patients (43.8%) (Fig. 3). Also, the 5-year survival rate of low BAF180 (8.1%) was significantly worse than that of high BAF180 patients (40.8%) (Fig. 3).

### Hazard analysis of SWI/SNF components in the patients treated with adjuvant gemcitabine

**Figure 4.**
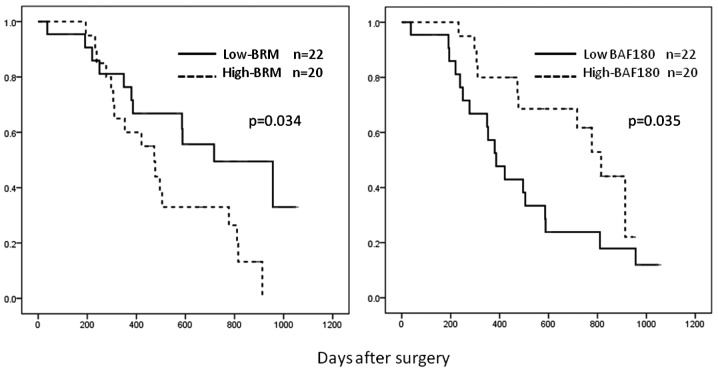
The survival curves of patients with adjuvant gemcitabine were compared by Kaplan-Maier method by the expression level of BRM and BAF180. The statistical significance was evaluated using log-rank test.

Multivariate analysis (Table V) and survival analysis (Fig. 4) showed that BRM-high and BAF180-low were independent prognostic factors for overall survival in the patients treated with adjuvant gemcitabine.

## Discussion

Chromatin remodeling factors have been the subject of great interest in oncology. However, little is known about their role in pancreatic cancer.

The SWI/SNF complexes are large, multi-subunit complexes containing 10 or more subunits, serving as a master switch that directs and limits the execution of specific cellular programs, such as differentiation and growth control (21). Each complex has one of the two different ATPase as core motor; BRM or BRG1, and subunits which are referred to as BAFs (BRM- or BRG1-associated factors). The BRM-containing complex is termed BRM/BAF. The BRG1-containing complexes are further divided into those that contain the BAF250a (termed BRG1/BAF) or the BAF180 (termed PBAF). These three types of complexes are believed to have different molecular functions (22).

There are several studies reporting that the subunit of SWI/SNF complex was decreased in cancer tissues. They revealed the mutation of *ARID1A*, which codes BAF250a protein, in about half of ovarian clear cell carcinomas (23,24), and *PBRM1*, which codes BAF180, in approximately 40% of renal cell carcinomas (25). Another study identified the SWI/SNF chromatin remodeling complex as tumor suppressor, by mediating retinoblastoma protein (RB)-derived regulation of the cell cycle (22,26,27). However, the roles of these subunits in pancreatic cancers are poorly understood.

In this study, we investigated the expression levels of 5 key subunits; BRM, BRG1, BAF250a, BAF180, which are the key subunits when subdividing complex types, and BAF47. There is established evidence that BAF47 is a tumor suppressor in rhabdoid tumors (28).

In the analysis of expression level and clinicopahological features, high BRM was related to worse clinicopathological features in general, including larger tumor size, T4 disease, other organ metastasis, lymphatic invasion, and stage IV disease. Stage IV disease was also correlated to high BRG1, which is reported to have similar biological function as BRM. On the other hand, better clinicopathological features were related to high BAF expression. High BAF180 was related to smaller tumor size, and high BAF47 was associated with negative lymphatic invasion.

In addition, our multivariate analysis revealed both high BRM and low BAF180 were independent prognostic indicators for poor survival, whereas the expression level of BRG1, BAF250a, and BAF47 were not related to overall survival.

As a next step, we investigated the prognostic significance of these factors in the patients with adjuvant gemcitabine. Gemcitabine remains standard therapy in the adjuvant and palliative settings for pancreatic cancer (29,30). However, the response rate of gemcitabine is very low, with only 18% of 1-year survival rate (31). Developing a novel biomarker, which predicts the response for gemcitabine, is urgently needed. In the analysis of the patients with gemcitabine, we reached the same result; both high BRM and low BAF180 were independent prognostic indicators for poor survival.

A previous study showed that BRM or BRG1 is lost in 10–20% of the bladder, colon, breast, esophageal, pancreatic and ovarian cancers by immunohistochemical staining of tissue microarrays (32). Another study reported BRM was lost in approximately 15–20% of primary non-small lung cancers, and silencing of BRM was a prognostic factor for poor outcome (33,34). Although BRM is supposed to be involved in many biological functions, these data showed BRM-containing complexes (BRM/BAF) as tumor suppressor in cancer tissue.

It is also reported that BRM has a role in trans cription of CD44 (35), which is important in the process of tumor-endothelium interactions, cell migration, cell adhesion, tumor progression and metastasis (36).

Our result showed that the patient with high BRM had a significantly worse survival than those without (5-year OS: 9.8 vs. 43.8%, p=0.009), suggesting BRM/BAF in pancreatic cancer may contribute to tumor progression.

We also revealed the significant relationship between high BAF180 expression and smaller-sized tumor, and identified BAF180 as an independent prognostic factor for better survival in pancreatic cancer.

BAF180 maps to the 3p12 region (37) where allele loss is frequent and homozygous deletion have been detected in lung and breast cancer cell lines (38,39). Thus, genes located on this region have been thought as candidates for tumor suppressors. Actually, it is reported that BAF180 mutation is associated with carcinogenesis of breast cancer, and BAF180 suppresses tumorigenesis through its ability to regulate p21 (40), which controls the cell cycle (41). Recent research also clarified BAF180 mutation in clear cell renal cell carcinoma (42). These results suggest the idea that BAF180-containing complexes (PBAF) suppress tumor progression, which does not contradict our present results.

BAF250a-containing SWI/SNF complexes (BRG1/BAF) are reported to have different structure and biological properties from PBAF (43,44). A previous study showed that BAF250a was deleted in as many as 30% of renal cell carcinoma and 10% of breast carcinoma (19,45). These results lead to the concept that BRG1/BAF appear to have antagonistic effect on cell cycle progression (46). However, our data did not show the relationship of BAF250a expression to clinicopathological features or overall survival in pancreatic cancer.

Based on this study, we reached the conclusion that high BRM, and low BAF180 are useful biomarker not only for the patients with curative resection, but also for those with adjuvant gemcitabine. Future investigation into biological functions of SWI/SNF components could lead to better management in pancreatic cancer.

## Figures and Tables

**Table I.** The clinicopathological characteristics of all patients.

Clinicopathological characteristics	No. of patients (n=68)
Age	
<65	30
≥65	38
Sex	
Male	36
Female	32
Tumor location in pancreas	
Head	46
Body/tail	22
Tumor size (cm)	
<4	29
≥4	39
Histological type	
Well/mod	32
Poor	36
T	
T1–3	38
T4	30
N	
N0	17
N1	51
M	
M0	53
M1	15
Curability of surgery	
R0	43
R1	25
Stage	
0–III	53
IV	15
Adjuvant gemcitabine	
Yes	42
No	26

Well, well differentiated adenocarcinoma; mod, moderately differentiated adenocarcinoma; poor, poorly differentiated adenocarcinoma.

**Table II.** Relation of SWI/SNF component expression to clinicopathological factors.

	BRM			BRG1			BAF250a			BAF180			BAF47		
Factors	Low	High	p-value	Low	High	p-value	Low	High	p-value	Low	High	p-value	Low	High	p-value
Age (years)															
<65/≥65	15/19	15/19	1.000	18/19	12/22	0.143	13/21	17/17	0.329	19/15	11/23	0.051	13/21	17/17	0.329
Gender															
Male/female	16/18	16/18	1.000	16/18	16/18	1.000	13/21	19/15	0.145	15/19	17/17	0.627	17/17	15/19	0.627
Tumor size															
<4/≥4 cm	19/15	10/24	0.027	12/22	17/17	0.220	14/20	15/19	0.806	10/24	19/15	0.027	15/19	14/20	0.806
Histology															
Well, mod/poor	18/16	14/20	0.331	11/23	21/13	0.015	14/20	18/16	0.331	13/21	19/15	0.145	15/19	17/17	0.627
T															
T1-3/4	25/9	13/21	0.003	23/11	15/19	0.051	17/17	21/13	0.329	19/15	19/15	1.000	20/14	18/16	0.625
N															
N0/N1	9/25	8/26	0.779	10/24	7/27	0.401	10/24	7/27	0.401	8/26	9/25	0.779	9/25	8/26	0.779
M															
M0/M1	30/4	23/11	0.041	27/7	26/8	0.770	24/10	29/5	0.114	25/9	28/6	0.380	28/6	25/9	0.380
Vessel invasion															
No/yes	12/22	8/26	0.287	11/23	9/25	0.595	7/27	13/21	0.110	10/24	10/24	1.000	8/26	12/22	0.287
Lymphatic invasion															
No/yes	15/19	6/28	0.018	13/21	8/26	0.189	9/25	12/22	0.431	9/25	12/22	0.431	15/19	6/28	0.018
Stage															
0–III/IV	18/16	5/29	0.001	17/17	6/28	0.005	10/24	13/21	0.442	11/23	12/22	0.798	14/20	9/25	0.200
Curability															
R0/R1	25/9	18/16	0.078	23/11	20/14	0.451	20/14	23/11	0.451	21/13	22/12	0.801	20/14	23/11	0.451

Well, well differentiated adenocarcinoma; mod, moderately differentiated adenocarcinoma; poor, poorly differentiated adenocarcinoma; inv, invasion.

**Table III.** Univariate analysis for overall survival in pancreatic cancer.

Factors	HR (95% CI)	p-value
Age (years)		0.035
<65	1.0	
≥65	0.533 (0.293–0.967)	
Sex		0.632
Male	1.0	
Female	0.865 (0.478–1.565)	
Tumor size (cm)		0.035
<4	1.0	
≥4	1.979 (1.048–3.739)	
Histology		0.002
Well/mod	1.0	
Poor	2.744 (1.429–5.271)	
T		0.071
T1–3	1.0	
T4	1.733 (0.955–3.146)	
N		0.602
N0	1.0	
N1	1.208 (0.594–2.458)	
M		0.010
M0	1.0	
M1	2.329 (1.222–4.439)	
Curability of surgery		0.020
R0	1.0	
R1	2.068 (1.121–3.815)	
BRM		0.011
Low	1.0	
High	2.225 (1.199–4.129)	
BRG1		0.601
Low	1.0	
High	0.853 (0.471–1.546)	
BAF250a		0.479
Low	1.0	
High	0.807 (0.446–1.461)	
BAF180		0.007
Low	1.0	
High	0.428 (0.231–0.793)	
BAF47		0.226
Low	1.0	
High	0.690 (0.378–1.258)	

HR, hazard ratio; 95% CI, 95% confidence interval; well, well differentiated adenocarcinoma; mod, moderately differentiated adenocarcinoma; poor, poorly differentiated adenocarcinoma.

**Table IV.** Multivariate analysis for overall survival in pancreatic cancer.

Factors	HR (95% CI)	p-value
Age		0.169
<65	1.0	
≥65	0.633 (0.330–1.214)	
Tumor size (cm)		0.755
<4	1.0	
≥4	1.122 (0.543–2.318)	
Histology		0.011
Well/Mod	1.0	
Poor	2.702 (1.253–5.830)	
M		0.486
M0	1.0	
M1	1.381 (0.557–3.424)	
Curability of surgery		0.076
R0	1.0	
R1	1.981 (0.932–4.214)	
BRM		0.032
Low	1.0	
High	2.144 (1.066–4.311)	
BAF180		0.041
Low	1.0	
High	0.501 (0.258–0.971)	

HR, hazard ratio; 95% CI, 95% confidence interval; well, well differentiated adenocarcinoma; mod, moderately differentiated adenocarcinoma; poor, poorly differentiated adenocarcinoma.

**Table V.** Multivariate analysis for overall survival in patients with adjuvant gemcitabine.

Factors	HR (95% CI)	p-value
Age		0.002
<65	1.0	
≥65	0.227 (0.089–0.580)	
Tumor size (cm)		0.280
<4	1.0	
≥4	0.593 (0.230–1.531)	
Histology		0.267
Well/Mod	1.0	
Poor	1.907 (0.610–5.964)	
M		0.923
M0	1.0	
M1	0.947 (0.315–2.847)	
Curability of surgery		0.784
R0	1.0	
R1	1.145 (0.433–3.029)	
BRM		0.017
Low	1.0	
High	3.411 (1.251–9.305)	
BAF180		0.016
Low	1.0	
High	0.336 (0.138–0.819)	

HR, hazard ratio; 95% CI, 95% confidence interval; well, well differentiated adenocarcinoma; mod, moderately differentiated adenocarcinoma; poor, poorly differentiated adenocarcinoma.

## References

[1] Parkin DM, Bray FI, Devesa SS (2001). Cancer burden in the year 2000. The global picture. Eur J Cancer.

[2] Jemal A, Siegel R, Ward E, Hao Y, Xu J, Thun MJ (2009). Cancer statistics, 2009. CA Cancer J Clin.

[3] Hidalgo M (2010). Pancreatic cancer. N Engl J Med.

[4] Reisman DN, Strobeck MW, Betz BL (2002). Concomitant down-regulation of BRM and BRG1 in human tumor cell lines: differential effects on RB-mediated growth arrest vs CD44 expression. Oncogene.

[5] Wade PA (2001). Transcriptional control at regulatory checkpoints by histone deacetylases: molecular connections between cancer and chromatin. Hum Mol Genet.

[6] Neely KE, Workman JL (2002). The complexity of chromatin remodeling and its links to cancer. Biochim Biophys Acta.

[7] Klochendler-Yeivin A, Muchardt C, Yaniv M (2002). SWI/SNF chromatin remodeling and cancer. Curr Opin Genet Dev.

[8] Davis PK, Brackmann RK (2003). Chromatin remodeling and cancer. Cancer Biol Ther.

[9] Narlikar GJ, Fan HY, Kingston RE (2002). Cooperation between complexes that regulate chromatin structure and transcription. Cell.

[10] Langst G, Becker PB (2001). Nucleosome mobilization and positioning by ISWI-containing chromatin-remodeling factors. J Cell Sci.

[11] Kingston RE, Narlikar GJ (1999). ATP-dependent remodeling and acetylation as regulators of chromatin fluidity. Genes Dev.

[12] Burns LG, Peterson CL (1997). The yeast SWI-SNF complex facilitates binding of a transcriptional activator to nucleosomal sites in vivo. Mol Cell Biol.

[13] Wu C (1997). Chromatin remodeling and the control of gene expression. J Biol Chem.

[14] Breeden L, Nasmyth K (1987). Cell cycle control of the yeast HO gene: *cis*- and *trans*-acting regulators. Cell.

[15] Recht J, Osley MA (1999). Mutations in both the structured domain and N-terminus of histone H2B bypass the requirement for Swi-Snf in yeast. EMBO J.

[16] Phelan ML, Sif S, Narlikar GJ, Kingston RE (1999). Reconstitution of a core chromatin remodeling complex from SWI/SNF subunits. Mol Cell.

[17] Tyler JK, Kadonaga JT (1999). The ‘dark side’ of chromatin remodeling: repressive effects on transcription. Cell.

[18] Wang W, Cote J, Xue Y (1996). Purification and biochemical heterogeneity of the mammalian SWI-SNF complex. EMBO J.

[19] Decristofaro MF, Betz BL, Rorie CJ, Reisman DN, Wang W, Weissman BE (2001). Characterization of SWI/SNF protein expression in human breast cancer cell lines and other malignancies. J Cell Physiol.

[20] McCarty KS, Miller LS, Cox EB, Konrath J, McCarty KS (1985). Estrogen receptor analyses. Correlation of biochemical and immunohistochemical methods using monoclonal antireceptor antibodies. Arch Pathol Lab Med.

[21] Bourgo RJ, Siddiqui H, Fox S (2009). SWI/SNF deficiency results in aberrant chromatin organization, mitotic failure, and diminished proliferative capacity. Mol Biol Cell.

[22] Reisman D, Glaros S, Thompson EA (2009). The SWI/SNF complex and cancer. Oncogene.

[23] Wiegand KC, Shah SP, Al-Agha OM (2010). ARID1A mutations in endometriosis-associated ovarian carcinomas. N Engl J Med.

[24] Jones S, Wang TL, Shih Ie M (2010). Frequent mutations of chromatin remodeling gene ARID1A in ovarian clear cell carcinoma. Science.

[25] Varela I, Tarpey P, Raine K (2011). Exome sequencing identifies frequent mutation of the SWI/SNF complex gene PBRM1 in renal carcinoma. Nature.

[26] Weissman B, Knudsen KE (2009). Hijacking the chromatin remodeling machinery: impact of SWI/SNF perturbations in cancer. Cancer Res.

[27] Roberts CW, Orkin SH (2004). The SWI/SNF complex - chromatin and cancer. Nat Rev Cancer.

[28] Biegel JA, Kalpana G, Knudsen ES (2002). The role of INI1 and the SWI/SNF complex in the development of rhabdoid tumors: meeting summary from the workshop on childhood atypical teratoid/rhabdoid tumors. Cancer Res.

[29] Heinemann V, Boeck S, Hinke A, Labianca R, Louvet C (2008). Meta-analysis of randomized trials: evaluation of benefit from gemcitabine-based combination chemotherapy applied in advanced pancreatic cancer. BMC Cancer.

[30] El-Rayes BF, Philip PA (2003). A review of systemic therapy for advanced pancreatic cancer. Clin Adv Hematol Oncol.

[31] Moore MJ, Goldstein D, Hamm J (2007). Erlotinib plus gemcitabine compared with gemcitabine alone in patients with advanced pancreatic cancer: a phase III trial of the National Cancer Institute of Canada Clinical Trials Group. J Clin Oncol.

[32] Glaros S, Cirrincione GM, Muchardt C, Kleer CG, Michael CW, Reisman D (2007). The reversible epigenetic silencing of BRM: implications for clinical targeted therapy. Oncogene.

[33] Reisman DN, Sciarrotta J, Wang W, Funkhouser WK, Weissman BE (2003). Loss of BRG1/BRM in human lung cancer cell lines and primary lung cancers: correlation with poor prognosis. Cancer Res.

[34] Fukuoka J, Fujii T, Shih JH (2004). Chromatin remodeling factors and BRM/BRG1 expression as prognostic indicators in non-small cell lung cancer. Clin Cancer Res.

[35] Batsche E, Yaniv M, Muchardt C (2006). The human SWI/SNF subunit Brm is a regulator of alternative splicing. Nat Struct Mol Biol.

[36] Martin TA, Harrison G, Mansel RE, Jiang WG (2003). The role of the CD44/ezrin complex in cancer metastasis. Crit Rev Oncol Hematol.

[37] Xue Y, Canman JC, Lee CS (2000). The human SWI/SNF-B chromatin-remodeling complex is related to yeast rsc and localizes at kinetochores of mitotic chromosomes. Proc Natl Acad Sci USA.

[38] Maitra A, Wistuba II, Washington C (2001). High-resolution chromosome 3p allelotyping of breast carcinomas and precursor lesions demonstrates frequent loss of heterozygosity and a discontinuous pattern of allele loss. Am J Pathol.

[39] Zabarovsky ER, Lerman MI, Minna JD (2002). Tumor suppressor genes on chromosome 3p involved in the pathogenesis of lung and other cancers. Oncogene.

[40] Xia W, Nagase S, Montia AG (2008). BAF180 is a critical regulator of p21 induction and a tumor suppressor mutated in breast cancer. Cancer Res.

[41] Harper JW, Adami GR, Wei N, Keyomarsi K, Elledge SJ (1993). The p21 Cdk-interacting protein Cip1 is a potent inhibitor of G1 cyclin-dependent kinases. Cell.

[42] Duns G, Hofstra RM, Sietzema JG (2012). Targeted exome sequencing in clear cell renal cell carcinoma tumors suggests aberrant chromatin regulation as a crucial step in ccRCC development. Hum Mutat.

[43] Nie Z, Xue Y, Yang D (2000). A specificity and targeting subunit of a human SWI/SNF family-related chromatin-remodeling complex. Mol Cell Biol.

[44] Sekine I, Sato M, Sunaga N (2005). The 3p21 candidate tumor suppressor gene BAF180 is normally expressed in human lung cancer. Oncogene.

[45] Wang X, Nagl NG, Flowers S, Zweitzig D, Dallas PB, Moran E (2004). Expression of p270 (ARID1A), a component of human SWI/SNF complexes, in human tumors. Int J Cancer.

[46] Nagl NG, Zweitzig DR, Thimmapaya B, Beck GR, Moran E (2006). The c-myc gene is a direct target of mammalian SWI/SNF-related complexes during differentiation-associated cell cycle arrest. Cancer Res.

